# Advanced Cervical Cancer: Leveraging the Historical Threshold of Overall Survival

**DOI:** 10.1055/s-0041-1728662

**Published:** 2021-04-15

**Authors:** Eduardo Paulino, Andreia Cristina de Melo

**Affiliations:** 1 Insituto Nacional do Câncer, Rio de Janeiro, RJ, Brazil; 2 Grupo Oncoclínicas, Rio de Janeiro, RJ, Brazil

Dear Editor,

Cervical cancer is a public health problem in low- and middle-income countries, where many patients are diagnosed at an advanced stage. After the Gynecology Oncology Group (GOG) 240 study, the first-line standard of care for patients in recurrent and/or metastatic settings includes the incorporation of bevacizumab with chemotherapy. Regarding the second line, no drug demonstrates a survival benefit and, therefore, no therapy can be considered the gold standard. The association of human papillomavirus (HPV) infection and immunosuppression with an increased risk of cervical cancer led to the hypothesis that the immune system may have an important role in this disease. More recently, pembrolizumab received Food and Drug Administration (FDA) approval as second-line therapy based on durable responses for patients with cervical cancer who expressed a combined positive score of  > 1%, although the response rate (RR) in this scenario was still poor (14%). 1

Over the past 3 decades, the GOG has studied many chemotherapeutic agents and has shown that the 12-month survival, RRs, and duration of response are low with chemotherapy alone. 2 Based on these findings, the 12-month survival has never increased beyond 30%, with RRs < 15%. Lan et al. 3 recently published in the Journal of Clinical Oncology the impressive results of camrelizumab, an antiprogrammed cell death-1 antibody (anti-PD1), plus apatinib, a tyrosine kinase inhibitor against vascular endothelial growth factor receptor-2 (anti-VEGFR-2) in 45 patients with advanced cervical cancer who progressed after at least 1 line of systemic therapy. This heavily pretreated population (57.8% received ≥ 2 lines of chemotherapy) showed RRs of 55.6%, and 12-month survival ∼ 60%. Of note, the median duration of the response and the median overall survival were not reached yet. 3 This combination compares favorably to each drug alone and highlights the exciting moment in cervical cancer research.

Important advances have been shown in the last decade with immunotherapy leveraging the 30% 12-month survival limit seen in historical studies by the GOG. Examples include vaccines (bioengineered modified listeria monocytogenes, Axalimogene filolisbac), anti-PD1 monotherapy (nivolumab, pembrolizumab, balsilimab) or combined with anti-cytotoxic T lymphocyte-associated protein 4 (anti-CTLA4), such as nivolumab plus ipilimumab and balstimab plus zalifrelimab. 1 4 5 6 7 Although the Axalimogene filolisbac vaccine showed a discouraging response rate of 2%, the 12-month survival reached 38%. 4 Monotherapy with nivolumab showed responses of 26%, and the 12-month survival reaching 77%; balsilimab demonstrated a RR of13% and a duration of response of 15 months. 5 7 Better results have been shown combining anti-PD1 with anti-CTLA-4. The combination of nivolumab (1 mg/Kg) plus ipilimumab (3 mg/Kg) showed a 12-month survival of 84% and a RR of 36% in previously treated patients; balstimab added to zalifrelimab showed a RR of 20%, with a median duration of response not achieved in previously treated patients. 6 7 This combination received fast track designation from the FDA.

A promising approach has also been demonstrated with the adoptive transfer of T lymphocytes. Stevanović et al. 8 showed that the infusion of tumor-infiltrating T cells resulted in two complete responses lasting 67 and 53 months at the time of publication. It is interesting to note that, although the tumor-infiltrating T cells were selected based on the reactivity of HPV 16 E6 and E7 oncoproteins, immunodominant T cell reactivities were directed against mutated neoantigens or a cancer germline antigen, rather than canonical viral antigens. 8

**Fig. 1:**
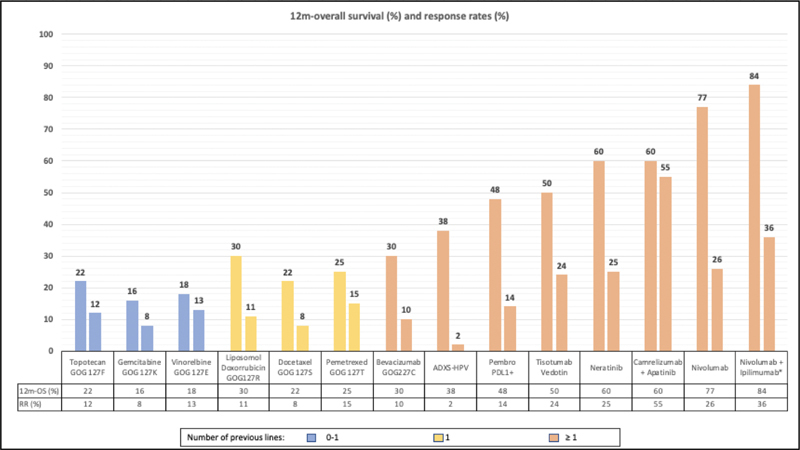
Summary of some recent compelling studies showing the evolution of 12-month survival and response rates. *Nivolumab 1 mg/Kg + ipilimumab 3 mg/Kg in previously-treated patients.

Impressive results have also been demonstrated in addition to immunotherapy. Examples include antidrug factor against tissue factor and antihuman epidermal growth factor receptor 2 (anti-HER2). 9 10 The tissue factor is overexpressed in cervical cancer. Tisotumab vedotin (antibody-drug conjugate against tissue factor) showed a RR of 24% and a 12-month survival ∼ 50%. 10 This drug has also been tested in combination with immunotherapy (NCT03786081). Human epidermal growth factor receptor 2 mutations are present in between 3 and 6% of cervical cancers according to sequencing studies. Neratinib, a pan-HER tyrosine kinase inhibitor, showed a RR of 25% and 12-month overall survival (12m-OS) of 60%. 9 Fig. 1 summarizes the 12m-OS evolution in the last 2 decades.

In the study by Lan et al., 3 only 22.2% of the population received bevacizumab previously. In the era of fast-growing evidence, immunotherapy combined (NCT03556839) or not (NCT03635567) with antiangiogenic agents is already being studied in a frontline, and even in combined curative chemoradiation for locally advanced tumors (NCT03830866, NCT04221945, NCT03833479). So, how would the camrelizumab/apatinib combination respond in this scenario?

In a post-hoc analysis, no difference in RR was observed between patients with PD-L1-positive and PD-L1-negative tumors. This finding goes in the opposite direction to that of Keynote 158, and is in line with the previously discussed studies that show RRs regardless of the expression of PD-L1, highlighting the importance of the search for a predictive biomarker for immunotherapy.

Treatment for advanced cervical cancer is an unmet need. Although we can clearly observe progress, < 20% of cancer discoveries touted as highly promising translates into clinical practice, 11 and the ongoing confirmatory phase III studies (NCT03257267) are essential to include immunotherapy as standard of care.
